# Functional Peptide-Based Biomaterials for Pharmaceutical Application: Sequences, Mechanisms, and Optimization Strategies

**DOI:** 10.3390/jfb17010037

**Published:** 2026-01-13

**Authors:** Dedong Yu, Nari Han, Hyejeong Son, Sun Jo Kim, Seho Kweon

**Affiliations:** College of Pharmacy, Chonnam National University, Gwangju 61186, Republic of Korea; yudedong@jnu.ac.kr (D.Y.); narih2804@jnu.ac.kr (N.H.); hyejeong1102@naver.com (H.S.)

**Keywords:** peptide-based biomaterials, drug delivery, cell-penetrating peptide, self-assembling peptide, peptide linker, sequence design, targeted delivery, AI

## Abstract

Peptide-based biomaterials have emerged as versatile tools for pharmaceutical drug delivery due to their biocompatibility and tunable sequences, yet a comprehensive overview of their categories, mechanisms, and optimization strategies remains lacking to guide clinical translation. This review systematically collates advances in peptide-based biomaterials, covering peptide excipients (cell penetrating peptides, tight junction modulating peptides, and peptide surfactants/stabilizers), self-assembling peptides (peptide-based nanospheres, cyclic peptide nanotubes, nanovesicles and micelles, peptide-based hydrogels and depots), and peptide linkers (for antibody drug-conjugates, peptide drug-conjugates, and prodrugs). We also dissect sequence-based optimization strategies, including rational design and biophysical optimization (cyclization, stapling, D-amino acid incorporation), functional motif integration, and combinatorial discovery with AI assistance, with examples spanning marketed drugs and research-stage candidates. The review reveals that cell-penetrating peptides enable efficient intracellular payload delivery via direct penetration or endocytosis; self-assembling peptides form diverse nanostructures for controlled release; and peptide linkers achieve site-specific drug release by responding to tumor-associated enzymes or pH cues, while sequence optimization enhances stability and targeting. Peptide-based biomaterials offer precise, biocompatible and tunable solutions for drug delivery, future advancements relying on AI-driven design and multi-functional modification will accelerate their transition from basic research to clinical application.

## 1. Introduction

Peptide-based biomaterials have emerged as versatile pillars in drug delivery, owing to their dual role as both therapeutic agents and high-performance pharmaceutical excipients. Unlike traditional excipients once deemed inert, modern peptide-based excipients are indispensable—they not only preserve API stability in vitro but also enhance in vivo stability, solubility, and bioavailability by acting as efficient carriers [1]. Their excellent biocompatibility, precise target specificity, and multifunctionality [2] address critical limitations of conventional excipients, such as poor biocompatibility and limited functional versatility.

**Figure 1:**
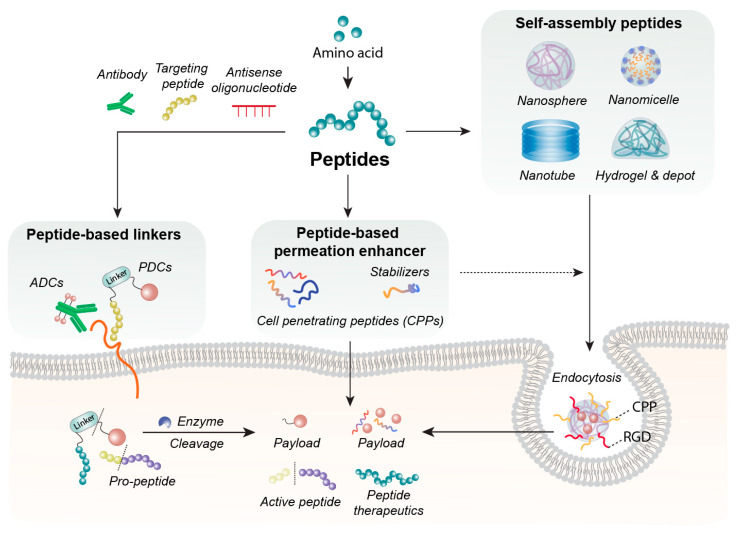
Applications using peptide-based biomaterials. Peptides composed of amino acids can self-assemble into diverse nanostructures, including nanospheres, nanomicelles, nanotubes, hydrogels, and depots, which serve as platforms for pharmaceutical delivery. Peptides also function as peptide-based permeation enhancer—such as cell-penetrating peptides or stabilizing agents—to enhance the cellular permeability or stability of therapeutic molecules. Furthermore, peptide-based linkers can be conjugated to antibodies, targeting peptides, or antisense oligonucleotides, where they are engineered to be cleaved by intracellular lysosomal enzymes such as cathepsins, enabling controlled payload release.

Currently, peptide-based excipients are widely integrated into drug delivery systems: they stabilize proteins and enzymes during production, freeze-drying, and storage [3], protect serum albumin in the bloodstream, and serve as surfactants or gelling agents to optimize formulation performance [4]. In this review, peptide-based excipients are categorized into three core functional types based on their roles: Peptide-based permeation enhancers (cell-penetrating peptides; CPPs and tight junction-modulating peptides; TJMPs), these target drug translocation-related biological sites—CPPs facilitate intracellular delivery via direct penetration or endocytosis, while TJMPs transiently modulate epithelial barriers—enhancing API delivery efficiency with favorable biocompatibility [5]. Self-assembling peptide carriers: The arrangement of hydrophilic and hydrophobic amino acids shapes peptide secondary structures and surface properties [6]. By engineering bioactive sequences and balancing amphiphilicity, these peptides self-assemble into micelles, hydrogels, or nanospheres to encapsulate poorly soluble APIs, enabling controlled release and protecting oral drugs from pH/enzymatic degradation [7]. Peptide-based targeted linkers: Applied in ADCs, PDCs, and prodrugs, these stimulus-responsive tethers covalently connect targeting modules and payloads [8]. Cleavable under specific physiological conditions (e.g., lysosomal acidity, tumor-overexpressed enzymes), they ensure systemic stability and on-demand site-specific drug release [3]. Figure 1 visually presents the framework for the multivariate application of peptide-based biomaterials in drug delivery.

Peptides’ inherent sequence tunability allows rational design of materials with optimized pharmacokinetics and targeting via structural modifications (e.g., cyclization, stapling) [9]. However, enormous sequence space, complex structure–activity relationships (SARs), and in vitro-in vivo discrepancies hinder manual optimization. Artificial intelligence (AI) technologies address these bottlenecks through high-throughput virtual screening, structure/activity prediction, and de novo sequence design [10].

This review systematically summarizes recent advances in the three categories of peptide-based excipients by focusing on studies published in the past five years (2020–2025), including their representative cases, core mechanisms, and distinctive characteristics. It further explores sequence-based optimization strategies, with a special emphasis on state-of-the-art AI-assisted design. Collectively, this work aims to provide actionable insights for the rational development of peptide-based biomaterials, accelerating their clinical translation.

## 2. Peptide-Based Permeation Enhancer

In recent years, peptide-based permeation enhancers have evolved from simple additive roles to multifunctional co-components in drug delivery. Biocompatible amino acid sequences with low immunogenicity can exert distinct permeation-enhancing effects, including cell penetration via cell-penetrating peptides (CPPs) and epithelial barrier modulation via tight junction modulating peptides (TJMP). The following section describes peptide-based permeation enhancers in the context of drug formulation optimization and permeation-promoting stabilization.

### 2.1. CPPs

CPPs are the predominant peptide-based excipients used for intracellular delivery. These peptides, typically comprising 5–30 amino acids, facilitate the entry of diverse molecular payloads into cells without compromising membrane integrity [9]. Such payloads may include proteins, nucleic acids, and nanoparticles [10]. CPPs were initially widely used due to their robust translocation capabilities, low cytotoxicity, and adaptability. Subsequently, they evolved into biomaterial carriers capable of targeted delivery and traversal of biological barriers [11].

Peptide-based CPPs are classified into cationic, amphiphilic, and hydrophobic categories according to their physicochemical features derived from sequence arrangement. Their uptake mechanisms are broadly categorized as direct penetration and endocytosis. Energy-free direct penetration involves various processes, including pore development, inverted micelle generation, and carpet-like models. Endocytic uptake occurs via macropinocytosis, clathrin-mediated endocytosis, and caveolin-mediated endocytosis, involving attachment to the cell membrane followed by vesicular internalization [12]. The peptide sequence, concentration, and cell type substantially influence uptake efficiency, making these parameters critical in CPP design.

*Cationic CPPs* carry positive charges at physiological pH due to the presence of basic amino acids such as arginine (pKa 12) and lysine (pKa 10.5). Due to the differences in pKa, arginine-rich peptides require higher scavenging to achieve equivalent charge repulsion than lysine-rich peptides, which is essential for maintaining the stability of their interaction with cell membranes [13]. Recent studies have shown that the guanidinium group of arginine is more critical for internalization than the peptide backbone, side chains, or linearity [14]. This is because the guanidinium group can form strong hydrogen bonds and electrostatic interactions with anionic components on the cell membrane, which serves as a key driving force for the initial binding of CPPs to the cell surface. The number and positioning are crucial for optimal interaction at physiological concentrations, Rational arrangement facilitates the binding of CPPs to cell membranes and promotes endocytosis. Studies have shown that 7–15 positively charged arginine and lysine residues can effectively enhance the subsequent endocytosis efficiency [15,16]. The internalization of cationic CPPs initiates with the interaction between their positively charged domains and the negatively charged phospholipids as well as heparan sulfate on the cell membrane via electrostatic forces, thereby anchoring the CPPs to the cell surface. Meanwhile, the negatively charged glycosaminoglycans on the cell membrane further modulate the endocytic uptake process by regulating the intensity and duration of the interaction between CPPs and membrane components, ultimately facilitating the transmembrane delivery of CPPs. The first CPP identified in 1988 was the Trans-Activator of Transcription (TAT peptide, GRKKRRQRRRPPQ), derived from the HIV-1 TAT protein. Multiple arginine residues adhere to heparan sulfate proteoglycans via electrostatic interactions, facilitating endocytosis [17]. The TAT peptide sequence (YGRKKRRQRRR), derived from HIV-1, functions as a receptor-independent CPP, efficiently delivering nanoparticles up to 200 nm into cells [18]. Branched dimer TAT (BTAT) structures exhibit up to 40-fold higher gene delivery efficiency in HeLa cells than monomeric TAT and promote reversible reductive dissociation for controlled cytosolic release [19]. TAT-decorated lipid nanoparticles maintain uptake efficiency under cold or metabolically inhibited conditions, supporting potential cold-chain distribution of vaccines or gene therapies [20]. The FDA has approved RTP004 peptides, 35-residue bi-CPP-containing peptides used in daxibotulinumtoxinA-lanm (DAXI) [21]. Rich in lysine and arginine, these peptides inhibit self-aggregation, enhance cell surface adsorption, increase neurotoxin attachment to presynaptic cells, and efficiently eliminate the intracellular botulinum toxin substrates. Polyarginines (R9), consisting of nine arginine residues, demonstrate approximately 20-fold greater uptake than TAT_49–57_ [22]. Studies of polyarginine (R*_n_*, *n* = 4–16) indicate that approximately eight residues achieve optimal translocation [23]. These peptides are currently used in drug conjugates, particularly to circumvent multidrug resistance mediated by p-glycoprotein. Triptolide has been formulated for delivery via disulfide bonds with R7, facilitating effective and stable penetration through the epidermis and dermis [8]. pVec (LLIILRRRIRKQAHAHSK) is an 18-amino acid peptide derived from the vascular endothelial cell cadherin protein. Although it contains lysine and arginine residues, replacing five hydrophobic amino acids at the N-terminus with L-alanine reduced cellular uptake [24]. Wortmannin, an endocytosis inhibitor, was used to elucidate the uptake mechanism. Additional cationic CPPs under investigation include P22N (RRRQRRKKRGY), DPV6 (R12) [25,26].

*Amphiphilic CPPs* contain both hydrophobic and hydrophilic domains, facilitating interactions with lipid bilayers via multiple uptake pathways. These peptides generally exhibit lower cytotoxicity than cationic CPPs, while achieving higher absorption rates [27]. Amphiphilic CPPs display primary, secondary (alpha-helix, beta-sheet), and proline-rich amphipathic properties. Primary amphipathicity consists of hydrophilic and hydrophobic domains interspersed with spacer sequences. Examples include the MPG peptide (GALFLGFLGAAGSTMGAWSQPKKKRKV) and Pep-1 peptide (KETWWETWWTEWSQPKKKRKV), which contain hydrophilic nuclear localization signals (PKKKRKV), in addition to hydrophobic domains and WSQP spacer motifs [28,29]. Secondary amphipathicity arises when hydrophobic residues align on one side and hydrophilic residues on the opposite side. Most alpha-helical structures exhibit one hydrophobic face and one polar or ionic area. Substituting residues with alanine to promote alpha-helix formation substantially enhances internalization [30]. The model amphipathic peptide (MAP, KLALKLALKALKAALKLA) demonstrates notable amphipathicity in alpha-helical structures [31]. Compared with the cationic CPP K21-PDP, MAP exhibited a 600-fold increase in small interfering RNA (siRNA) absorption after 6 h, comparable to Lipofectamine 2000 [32]. The TP10 sequence (AGYLLGKINLKALAALAKKIL), comprising 21 amino acids, generates positively charged helical structures that penetrate phospholipid membranes in monomeric form without creating pores [33]. This peptide exemplifies the relationship between intrapeptide charge distribution, structural properties, and biological activity. Penetratin (RQIKIWFQNRRMKWKK) infiltrates cells owing to its structural flexibility, enabling the formation of either alpha- or beta-sheet structures depending on environmental conditions [34]. The hydrophobic core residues W6 and F7 substantially affect structural cellular penetration. Antp(43–58) adopts an alpha-helical conformation; however, its assembly is modulated by small conformers at the termini, with alpha-helix content increasing in proximity to membranes [35]. In contrast, the cyclic variant of the same sequence exhibits negligible cellular absorption, indicating that the internalization of amphiphilic CPPs is strongly dependent on their structural conformation [36]. Transportan (GWTLNSAGYLLGKINLKALAALAKKIL) is a 27-amino acid chimeric cell-penetrating peptide derived from the N-terminal sequences of galanin and mastoparan. This peptide has facilitated the development of the CPP series, including PepFect and NickFect, which form nanocomplexes and nanoassemblies [37]. The sweet arrow peptide (SAP, VRLPPPVRLPPPVRLPPP), characterized by a polyproline motif, comprises 50% proline content and forms a polyproline II (PP II) structure. This arrangement positions hydrophobic residues on one side and hydrophilic residues on the opposite side, producing secondary amphipathicity [38]. D-amino acid self-assembling peptide (D-SAP), the D-amino acid variant, exhibits enhanced serum stability while maintaining internalization efficiency, highlighting the benefits of chiral secondary structures [39].

*Hydrophobic CPPs* primarily consist of nonpolar amino acid residues, exhibit low net charge, and contain fewer than 20% cationic amino acids. To mitigate toxicity associated with highly positively charged peptides, hydrophobic motifs and augmented hydrophobic segments are employed to enhance membrane interactions [40]. The incorporation of tryptophan (Trp) in DNA delivery demonstrates the importance of moderately hydrophobic functional groups, whereas hydrophilic residues maintain solubility in serum [5]. Nevertheless, hydrophilic amino acids are included to preserve solubility in serum. Hel13-5 (KLLKLLLKLWLKLLKLLL), which comprises hydrophobic leucine and hydrophilic lysine residues, effectively facilitates DNA delivery owing to its high hydrophobic content [41]. Hydrophobic motifs occasionally play a crucial role in peptide aggregate formation, influencing cellular uptake and inhibiting DNA degradation, thereby rendering them valuable as nanostructural materials and functional components. PFVYLI, a hexapeptide from the C-terminal region of α1-antitrypsin, exhibits nucleus-targeting properties with minimal cytotoxicity. These peptides contribute to the formation of nanostructural materials, including nanofibers, via gene delivery and self-assembly [42]. Mastoparan (INLKALAALAKKIL), modified with a single acyl chain, dialkylcarbamoyl group, or cholesteryloxycarbonyl group, and the alpha-helix model peptide (LARLLARLLARL) modified with a single acyl chain, retain alpha-helix structures and facilitate self-assembly [43]. Pept1 (PLILLRLLRGQF), Pept2 (PLIYLRLLRGQF), IVV-14 (KLWMRWYSPTTRRYG), and Pep-7 (SDLWEMMMVSLACQY) exhibit hydrophobic alpha-helix structures with enhanced permeability and tumor cell penetration [44,45,46]. Table 1 shows a summary of representative examples of CPPs along with their corresponding sequences.

### 2.2. TJMP

**Figure 2:**
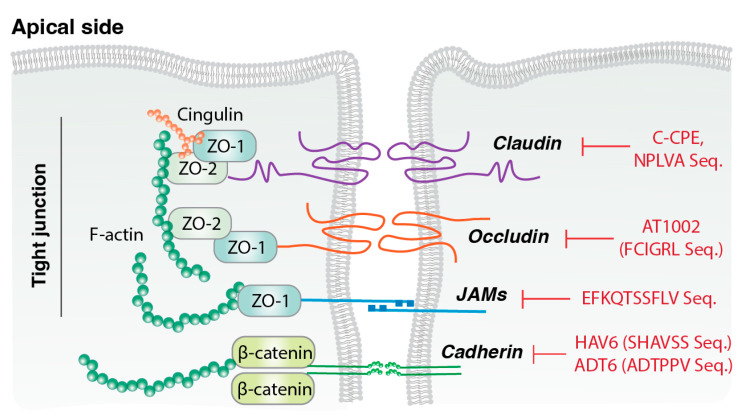
Mechanisms of action of TJMPs. Peptide-based tight junction-modulating peptides (TJMPs) that inhibit the mechanisms of key tight junction proteins, including claudins, occludins, and junctional adhesion molecules (JAMs). These peptides act by disrupting protein–protein interactions or signaling pathways that maintain tight junction integrity, thereby transiently enhancing paracellular permeability.

Tight junctions seal the intercellular space between adjacent epithelial or endothelial cells and primarily regulate the absorption of chemicals, solutes, water, and ions, while controlling pathogen translocation and maintaining paracellular permeability. As shown in Figure 2 tight junction proteins are essential for maintaining the structural integrity and barrier function of epithelial and endothelial cell layers and also participate in intracellular signaling pathways that regulate cell proliferation, differentiation, and immune responses [50]. Because of their selective permeability, these proteins are critical for fluid balance and nutrient transport. Claudins are major transmembrane components of tight junctions, with more than 20 identified isoforms. They are key regulators of paracellular barrier formation and selective pore creation, and tissue-specific claudins determine intercellular permeability characteristics [51]. Occludin, a 65 kDa transmembrane protein, contributes to barrier integrity and signal transmission [52]. Junction adhesion molecules (JAMs) are single-transmembrane, immunoglobulin-like proteins involved in intercellular adhesion and leukocyte migration. Zonular Occludens (ZO) proteins (ZO-1, ZO-2, ZO-3) link transmembrane proteins (claudins, occludins, JAMs) to the actin cytoskeleton, and support tight junction assembly, cell migration, proliferation, and barrier formation [53]. In drug delivery, TJMPs transiently and reversibly alter epithelial barriers to enhance drug absorption, particularly for hydrophilic compounds and macromolecules that otherwise display limited permeability [54]. These peptides function by interacting with specific tight junction proteins.

*ZO-derived peptides:* The synthetic peptide AT1002 (FCIGRL), derived from the zonula occludens toxin, transiently and reversibly opens tight junctions, facilitating drug absorption. The mechanism of AT1002 involves inducing actin microfilament rearrangement, which reversibly opens tight junctions through multiple pathways, thereby transiently increasing epithelial permeability and enhancing paracellular transport. This transient permeability enhancement facilitates the absorption of diverse compounds across intestinal, nasal, tracheal, or transdermal routes, ultimately improving their bioavailability. Notably, AT1002’s interaction with the zonulin receptor does not induce cytotoxicity or long-term epithelial damage, and preserve barrier integrity after transient permeability enhancement [55].

*Claudin-specific modulators:* Tight junction modulators that act on claudins allow precise control over paracellular permeability. The CIC2 peptide, derived from claudin-1, modulates barrier function by interacting with claudin-1 and is internalized into vesicular structures that colocalize with claudin-1 [56]. The C-terminal fragment of *Clostridium perfringens* enterotoxin (C-CPE) is a well-characterized claudin-targeting protein that binds claudin-3 and claudin-4, promoting their lateral cis-assembly and disassembly of tight junctions [57]. This activity results in concentration-dependent decreases in transepithelial electrical resistance (TEER) and enhances the permeability of macromolecules, proteins, and antibodies. C-CPE is particularly useful for targeting tissues or tumors that overexpress claudins, such as claudin-4 in pancreatic cancer. Its 12-residue binding motif, which includes the essential NPLVA sequence, disrupts claudin formation and facilitates junctional breakdown [58]. Recent C-CPE fusion proteins have been applied in diagnostic imaging and the study of tight junction biology. Claudin-5 binding peptides, including claudin-5 (CLDN5) extracellular loop-derived peptides f1-C5C2 and f3-cCPE-mut1, modulate blood–brain barrier (BBB) permeability and play critical roles in the regulation of intercellular transport across the BBB [59,60].

*E-cadherin-derived peptides:* E-cadherin is a calcium-dependent cell adhesion protein essential for maintaining intracellular tight junction integrity. E-cadherin–derived peptides modulate barrier permeability in tissues such as the intestinal epithelium and BBB [61]. The extracellular domain consists of five subdomains (EC1–EC5) that are responsible for cell–cell adhesion and drug permeability. The EC1-derived peptide HAV6 (Ac-SHAVSS-NH_2_) and the EC1/EC2-derived ADT-6 peptide (Ac-ADTPPV-NH_2_) interact with E-cadherin at the epithelial barriers and facilitate paracellular penetration of anticancer agents [62]. The HAV motif interferes with zipper-like adhesive interactions, transiently reducing adhesion and increasing permeability. HAV/ADT peptides and related bulge motif peptides (BLG2, BLG4) inhibit junctional resealing, prolonging paracellular opening [63]. Cyclic analogs, including cyclic Hepatitis A virus cell-adhesion peptide 3 (cHAVc3) and acetylated dimeric tight junction–modulating peptide 5 (ADTC5), enhance proteolytic resistance and improve stability for oral delivery [64]. E-cadherin-targeting peptides provide selective control of paracellular permeability with minimal off-target effects and enhance BBB transport. These peptides also synergize with anticancer agents to inhibit tumor cell adhesion and metastasis [65].

## 3. Self-Assembling Peptides in Drug Formulation

**Figure 3:**
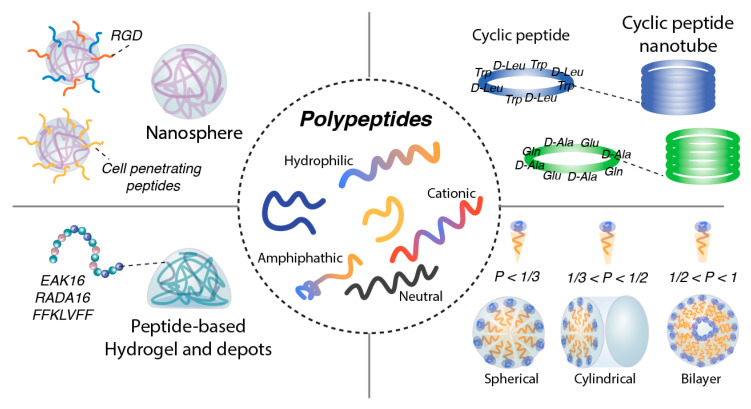
Peptide-based self-assembling nanostructures. Hydrophilic, cationic, neutral, or amphipathic polypeptides can self-assemble into nanospherical structures. Their surfaces can be further modified with targeting motifs such as RGD peptides or cell-penetrating peptides to enhance cellular uptake. Cationic or anionic peptides are also capable of forming peptide-based hydrogels and depot systems. In addition, cyclic peptides can assemble into cyclic peptide nanotubes through directional hydrogen bonding. Peptide micelles exhibit diverse morphologies—including spherical, cylindrical, or bilayer structures—depending on their length and degree of hydrophobicity.

Self-assembling peptides are short amino acid sequences that spontaneously organize into ordered supramolecular structures through non-covalent interactions under specific environmental conditions. As shown in Figure 3 these assemblies can form diverse architectures, including nanoparticles, microparticles, micelles, hydrogels, nanoscaffolds, and nanofibers [66,67,68,69,70,71,72]. Owing to their inherent tunability, biocompatibility, and biodegradability, self-assembling peptides have attracted considerable interest as versatile platforms for biomedical applications, including drug delivery carriers, scaffolding materials for tissue engineering, and functional components for diagnostic probes.

Among these diverse self-assembled architectures, amyloid fibrils stand out as an interesting and representative case of peptide self-assembly. They are fibrous aggregates rich in cross-β-sheet structures formed by peptide or protein misfolding. Initially used to describe pathological deposits associated with amyloidosis and neurodegenerative diseases, the term “amyloid fibrils” is now widely defined as any peptide/protein that forms cross-β-sheet fibrils, regardless of pathological relevance [73] Structurally, peptide β-strands first assemble into parallel or antiparallel β-sheets (β-layers), which further construct cross-β structures; in these configurations, β-strands are oriented perpendicular to the fiber axis and eventually elongate to form amyloid fibrils. These fibrils maintain high structural stability through extensive hydrogen bonding and exhibit structural diversity due to differences in amino acid sequences and environmental conditions—a phenomenon that closely correlates with the driving forces of peptide self-assembly [74].

At the molecular level, peptide assembly is driven by a combination of non-covalent interactions and intrinsic physicochemical properties. The non-covalent interactions encompass hydrogen bonding involving serine (Ser), threonine (Thr), tyrosine (Tyr), electrostatic interactions between positively charged residues lysine (Lys), arginine (Arg) and negatively charged residues aspartic acid (Asp) and glutamic acid (Glu) [75,76,77]; hydrophobic interactions involving valine (Val), leucine (Leu), isoleucine (Ile), phenylalanine (Phe) and tryptophan (Trp) [78]; π–π stacking interactions involving Phe, Tyr, Trp [76,79]; and van der Waals forces.

A core component of the intrinsic physicochemical properties lies in the peptide backbone, which serves as the fundamental scaffold for the formation of secondary structures such as α-helices and β-sheets—key intermediates in self-assembly. Its atomic composition, conformational flexibility, modification methods, and dynamic transitions directly determine the type, stability, and functional adaptability of these secondary structures. The C=O and NH atoms of the backbone can form specific side chain-independent non-covalent interactions, constituting the “seed structures” for secondary structure formation and laying the foundation for the initiation and extension of folding in unfolded peptide chains [80]. Artificial regulation of secondary structures can be achieved through modifications to the Cα, N atoms, or amide bonds of the backbone: N-substitution modifications (such as methylation and alkylation) reduce the hydrogen bond participation capacity of the backbone NH, disrupting the stability of α-helices and β-sheets to enhance peptide solubility; in contrast, introducing alkyl or aryl substituents at the Cα position can stabilize the β-hairpin conformation through steric effects and promote β-sheet stacking [81]. Meanwhile, the conformational plasticity of the peptide backbone enables it to respond to external stimuli such as pH, enzymes, and ionic strength, realizing the “dormancy-activation” transition of secondary structures—for example, the backbone of cationic antimicrobial peptides adopts a random coil conformation due to electrostatic repulsion under physiological pH, while in an acidic environment, backbone protonation triggers φ, ψ angle rearrangement, forming α-helices and activating membrane penetration ability [82]. Furthermore, the peptide backbone can mediate the connection of different secondary structures through conformational continuity; the amphiphilicity endowed by the backbone renders α-helices with facial amphiphilicity and β-sheets with sheet-like amphiphilicity, further driving the assembly of secondary structures into supramolecular structures such as nanofibers and hydrogels, completing the transition from basic structures to functional materials.

Intrinsic properties influencing assembly comprise amino acid sequence composition, secondary structural propensity, charge distribution, peptide length, and chemical modifications. Notably, variations in these driving forces under different environmental conditions result in distinct architectures and functional outcomes.

By strategically combining amphiphilic, charged, and aromatic residues, self-assembling peptide systems can be tailored to produce nanostructures and functionalities spanning the nanoscale to the microscale. Self-assembling peptide structures with dimensions of 10–200 nm can be engineered to deliver therapeutic or diagnostic agents to targeted sites. Depending on the peptide design and assembly conditions, these structures may assume various forms, including nanospheres, nanotubes, nanocapsules, nanofibers, and nanovesicles. In the following sections, the applications and limitations of peptide-based nanospheres, nanotubes and nanofiber, nanotubes and nanofiber, hydrogels and depots in drug delivery, tissue engineering, and diagnostics are discussed.

### 3.1. Peptide-Based Nanospheres

Peptide-based nanospheres are a class of zero-dimensional (0-D) nano-architectures formed by the self-assembly of peptide molecules through non-covalent interactions [83], exhibiting regular spherical or near-spherical morphologies. Their core characteristic is a “non-directional, spherical aggregate morphology,” which can be divided into two types: solid spherical aggregates and porous spherical frameworks. A notable advantage of peptide nanospheres is the tunable physicochemical properties—size, charge, and hydrophobic-hydrophilic balance—which can be precisely engineered to overcome biological barriers [84]. Nanospheres smaller than 100 nm can penetrate dense tissue, such as the ECM, and access regions inaccessible to larger microspheres. These nanoscale assemblies facilitate enhanced molecular diffusion and cellular uptake, including translocation across tight junctions and vascular endothelium. Once in circulation, nanospheres accumulate in tumors via the enhanced permeability and retention (EPR) effect and release encapsulated drug through slow diffusion or environmentally triggered disassembly [85].

Relevant amino acid residues can stabilize the spherical conformation by forming hydrogen bond networks and hydrophobic interactions. For example, L,L-cyclic peptides containing hydrophobic (Trp), cationic (Arg), and cysteine (Cys) residues serve as building blocks, which self-assemble into peptide-based nanospheres via these synergistic interactions and function as intracellular delivery carriers for VEGF siRNA/ASO [86]. In contrast, for aromatic amino acid-containing peptides, π–π stacking and electrostatic interactions are the primary driving forces for nanosphere construction. A typical illustration is the single-chain peptide EOG-10, which triggers the self-assembly of the triple-helical peptide A-PRG through electrostatic interactions, leading to the formation of a paired “side-by-side” binding mode. This intermediate structure is further cross-linked and stabilized by π–π stacking, ultimately yielding nanosphere architectures [87].

Recently, a novel self-assembly process—dissipative self-assembly (DSA)—has been reported, in which sonically driven DSA yields supramolecular nanoclusters [88]. The transient energy input (cavitation) provided by ultrasound pushes aromatic amino acids (e.g., tryptophan) into a high-energy state while creating a transient liquid-air interface. Through this method, not only is the rapid and uniform assembly of nanospheres achieved, but the resulting nanoparticles also possess unique optical properties and biological functions—rendering them suitable for investigating the intracellular degradation of nanoparticles, the sustained release of anticancer drugs, and the detection of intracellular transport in living cells.

Furthermore, the surface of peptide nanospheres is rich in amino acid reaction sites, can be functionalized with targeting ligands, such as the RGD motif or antibody fragments, to achieve selective delivery to specific cells or tissues. For example, peptides containing the RGD tumor-targeting motif (VVVVVKKGRGDS) self-assemble into micelles with a hydrophobic Val core and positively charged Lys residues, exhibiting pH-dependent responsiveness [89].

Peptide-based nanospheres can traverse physiological barriers, such as the BBB, via receptor-mediated transcytosis, particularly when chemically functionalized for improved targeting and serum stability [90]. CPPs such as TAT, RVG, and angiopep-2 facilitate the transport of cargo across the BBB, enabling the delivery of neurological drugs and imaging agents previously limited by restricted access.

Beyond the BBB, these nanospheres efficiently traverse mucosal barriers, tumor stroma, and vascular endothelium, while their self-assembling nature endows them with environmentally triggered transformation capabilities—responding to pH, ionic strength, or enzymatic activity—to achieve selective payload release at pathological sites.

Notably, in cancer therapy, peptide-based nanospheres not only enhance uptake by cancer cells and overcome multidrug resistance but also achieve deep tumor penetration through targeted functionalization. For example, peptides containing the ‘CendR’ motif, such as tLyP-1 (CGNKRTR), bind to neuropilin-1 (NRP-1)—a receptor involved in tumor angiogenesis and metastasis—exhibiting tumor-specific tissue penetration and up to 4.8-fold increased tumor accumulation, as validated by SPECT/CT imaging [91]. Fusion of tLyP-1 to human ferritin nanospheres further augments cellular uptake and tumor penetration, while NRP-1 targeting in CD8^+^ T cells enhances PD-1 blockade efficacy and promotes long-term immune memory [92].

The unique advantages of peptide-based nanospheres have been well exploited in the construction of theranostic platforms, for example cyclic peptide [(WR)_5_C] and aqueous GdCl_3_ solution are combined via an in situ one-pot method to form nanoparticles [93]. These peptide-Gd nanoconstructs not only exert gadolinium-mediated tracing effects (enabling MRI imaging) but also enhance cellular uptake and improve biocompatibility through the peptide-based nanosphere scaffold. When further loaded with other therapeutic agents, they can synergistically achieve robust therapeutic efficacy.

Another intriguing case involves PEGylated tetrapeptide (PEG6-Y4) as the building block [94]. At room temperature, PEG6-Y4 spontaneously self-assembles into non-fluorescent, water-soluble fibrous aggregates. Upon high-temperature treatment, it undergoes Tyr-Tyr coupling reactions, triggering a structural transition into fluorescent nanospheres. Notably, these nanospheres exhibit unexpected photoluminescence in the blue-green visible light region (440–480 nm), endowing them with fluorescence tracing potential and thus holding promise as a safe and effective biodiagnostic tool.

Table 2 summarizes representative examples of peptide-based nanospheres employed as functional excipients to enhance drug delivery, self-assembling peptide-based nanospheres—featuring tunable structures and facile modifiability—emerge as next-generation theranostic platforms that combine superior drug-delivery performance with multimodal biological functions. Their ability to traverse physiological barriers and penetrate deep tumor tissues positions them as promising tools for precision medicine.

Despite these advantages, peptide nanocarriers present several limitations. Foremost among these is poor stability in complex physiological environments, as non-covalent assemblies readily dissociate or aggregate under dilution, pH fluctuations, or interaction with serum proteins [95]. Proteolytic enzymes further accelerate degradation, reducing circulation time; chemical modifications, such as the incorporation of D-amino acids or cyclization, mitigate enzymatic susceptibility [96]. Another limitation is the challenge of controlling size and morphology, as minor variations in sequence or environmental conditions can result in unpredictable assembly outcomes, posing difficulties for scale-up manufacturing and reproducibility [97]. Although high drug loading can be achieved, weak drug–carrier interactions may necessitate the administration of large payload doses. Furthermore, reports of potential immunogenicity and rapid clearance by the immune system have prompted the incorporation of stealth polymers and responsive stability designs to enhance circulation [66]. Ultimately, while peptide nanocarriers offer substantial promise, ongoing optimization is required to ensure their integrity and functional performance.

### 3.2. Cyclic Peptide Nanotubes and Nanofiber

Cyclic peptide nanotubes represent peptide stacking architectures stabilized by hydrogen bonding. Heterochiral cyclic peptides, consisting of alternating D- and L-amino acids, adopt extended β-sheet-like conformations that stack to form hollow cylindrical nanotubes. Nanotubes generated using the cyclo[(D-Ala-Glu-D-Ala-Gln)_2_] sequence exhibit internal diameters of 7–8 Å, with the diameter tunable by varying the number of amino acids in the peptide ring [98]. Assembly of these structures is pH-dependent: acidic environments favor stacking, whereas alkaline environments inhibit assembly due to electrostatic repulsion between negatively charged glutamic acid residues. Cyclic peptide nanotubes display excellent ion transport properties and selectivity. For example, the cyclo[(Trp-D-Leu)_4_-Gln-D-Leu] structure demonstrated potassium and sodium ion conductivities of 65 pS and 55 pS, respectively, surpassing those of gramicidin A [99]. These nanotubes exhibit pronounced cation selectivity, with conductivity modulated according to the lyotropic series from lithium to cesium ions. In drug delivery applications, cyclo[(Trp-D-Leu)_4_-Gln-D-Leu] nanotubes have been explored for the delivery of 5-fluorouracil [100]. Molecular dynamics simulations indicate that drug molecules traverse the inner channel of the nanotube via a hopping mechanism, interacting with the hydrophobic interior and passing through the terminal kink regions for release.

Stable nanofiber production in aqueous peptide solutions supports the delivery of hydrophobic molecules. These peptides typically contain four domains: (1) hydrophobic tails for self-assembly, (2) charged amino acids for solubility, (3) sequences that mediate intramolecular hydrogen bonding, and (4) functional epitopes for biological activity. Modulating these domains allows control over self-assembly behavior and drug release properties [101,102]. Biocompatible and biodegradable peptide amphiphiles decompose into standard amino acids and hydrophobic fragments, reducing concerns regarding long-term accumulation and toxicity.

Furthermore, peptide nanotube architectures offer advantages as ion channel models, platforms for membrane protein research, and selective drug delivery systems. Key strengths include precise diameter control via sequence engineering, robust structural stability, and selective permeability [103]. However, challenges persist in terms of synthetic complexity and the restricted range of achievable nanotube sizes. Cyclic peptide structures can self-assemble through hydrogen bonding, forming hollow nanotubes. Beginning with octapeptides, these nanotubes exhibit notable stability and tunable internal diameters, rendering them suitable as drug carriers and molecular transport channels. For instance, FF-DOX nanotubes loaded with doxorubicin have shown enhanced cytotoxicity against drug-resistant cancer cells, highlighting the efficiency of self-assembly drug delivery [104].

### 3.3. Nanovesicles and Micelles

Certain amphiphilic peptides, which contain both hydrophobic and hydrophilic segments, can self-assemble into nanospheres capable of encapsulating drugs. These structures are analogous to polymeric micelles but are composed entirely of peptides or peptide–lipid conjugates. Examples include peptosomes (peptide-based liposomes) and polymeric peptide micelles [105]. These peptides exhibit surfactant-like behavior, forming aggregates such as micelles, microparticles, and nanoparticles in aqueous environments through the organization of a hydrophobic core surrounded by a hydrophilic shell. For example, lipid-mimetic peptide sequences such as A6D (AAAAAD) and V6K (VVVVVK) utilize repeated hydrophobic residues, including alanine (Ala) and Val, to mimic the fatty acid tail, while Asp and Lys function analogously to the polar head groups of lipids [106]. These peptides undergo self-assembly above a critical concentration, enabling the encapsulation of poorly soluble drugs within the hydrophobic core of the resulting structures. This assembly enhances the solubility and stability of the payload, rendering these constructs effective carriers for drug delivery. Based on the molecular packing parameter (*P*), micelles are classified as spherical (*P* < 1/3), cylindrical (1/3 < *P* < 1/2), flexible bilayers, or vesicles (1/2 < *P* < 1) [107]. Notably, peptide-based vesicles (nanovesicles) stand out as a distinct and functionally prominent type, they are hollow spherical structures formed by amphipathic molecules assembling into bilayer membranes, creating internal spaces for encapsulating drugs and other molecules. Vesicle-to-micelle transitions (VMTs) are stimulus-responsive processes triggered by reactive oxygen species (ROS), light, or heat, enabling controlled drug release [108].

Stimulus-responsive vesicle–micelle systems have been extensively developed. In catanionic vesicle systems responsive to ROS, a mixture of (4-phenylthiophenyl)diphenyl-sulfonium triflate (PDST) and sodium dodecylbenzene sulfonate (SDBS) forms vesicles that transition to micelles upon exposure to hydrogen peroxide (H_2_O_2_), with PDST’s thioether oxidized to hydrophilic sulfoxide [108]. This transition is monitored via turbidity, light scattering, and cryo-TEM. Additionally, temperature-induced VMTs have been reported, in which increased temperature converts vesicles into micelles, depending on hydrophobic tail length [109]. Studies involving bile salts (sodium cholate, sodium deoxycholate) demonstrate that their amphiphilic structure and large headgroup area promote the vesicle–micelle transition by increasing the average surfactant headgroup area [110].

In addition to these single-chain amphipathic peptide systems, a class of structurally engineered peptides with more sophisticated architectures—Gemini surfactant-like peptides (GSLPs)—has garnered increasing attention for advanced drug delivery applications. Gemini surfactant-like peptides (GSLPs) exhibit dimeric architectures in which two hydrophobic or self-assembling domains are connected by flexible, charged spacers. The GNNQQNY-PKKP-GNNQQNY peptides, for instance, combine the GNNQQNY and beta-sheet-forming sequences with PKKP linkers that are flexible and hydrophobic [111]. These peptides have low critical assembly concentrations and self-assemble into nanocages, enabling high drug loading and solubilization. Extended sequences may exhibit stability limitations [112]. The APK peptide, containing two prolines and turn-forming units, demonstrates enhanced self-assembly, effectively encapsulates diverse hydrophobic drugs, and exhibits antibacterial activity [113].

Amphiphilic peptides also serve as protein and peptide stabilizers by generating surface-active interactions at air-water or oil-water interfaces. These interactions inhibit protein aggregation, unfolding, and proteolytic degradation, while promoting surface adsorption and solubilization, providing a safer alternative to conventional surfactants [114]. Arginine-rich peptide stabilizers contain multiple arginines that stabilize protein and peptide formulations, thereby mitigating aggregation in biopharmaceuticals. RD2 peptides exemplify arginine-rich aggregation inhibitors that suppress amyloid-beta aggregation in Alzheimer’s disease. Their high net positive charge (+6.2) interacts with negatively charged surfaces, reduces hydrophobic interactions, and facilitates refolding and stabilization of membrane proteins [115].

Table 3 presents representative examples of nanovesicles and micelles. Both vesicle and micelle structures can encapsulate hydrophilic or hydrophobic drugs, genes, or cosmetic ingredients. Their resemblance to biological membranes confers high biocompatibility, and their responsiveness to stimuli (ROS, light, heat) allows for tunable drug release. However, structural instability, leakage of encapsulated contents, and challenges in achieving uniform size remain substantial technical hurdles.

### 3.4. Peptide-Based Hydrogels and Depots

Peptide-based hydrogels are soft, water-rich materials formed through the network assembly of peptide nanofibers. Individual peptide molecules self-assemble into one-dimensional fibrils, often adopting β-sheet-rich arrangements, forming three-dimensional meshes that retain more than 90% water content. Supramolecular hydrogels composed of peptides and water can be triggered to gelate by environmental cues, such as changes in pH, ionic strength, temperature, or enzymatic activity [116]. Mechanistically, gelation proceeds through a multi-step process in which peptides form secondary structures (e.g., α-helices and β-hairpins), aggregate into thin nanofibers under specific conditions, and ultimately yield macroscopic gels over time.

The formation of these peptide hydrogels is primarily driven by two typical assembly mechanisms, depending on the amino acid composition of the peptides. First, peptides composed predominantly of charged residues (Ser, Thr, Tyr, Lys, Arg), such as EAK16 and RADA16, form β-sheet arrangements in aqueous solution via a zipper-like assembly mechanism [117]. The resulting structures are stable nanofibers or nanoscaffolds that can further organize into hydrogel networks, with RADA16-based hydrogels standing out for their biological versatility, particularly in tissue engineering and functional composite systems. RADA16-I (Ac-(RADA)4-NH_2_) self-assembles into nanofiber hydrogels that closely resemble the extracellular matrix (ECM), This ECM-mimetic property not only enables 3D cell culture systems [118] but also makes RADA16-based hydrogels ideal scaffolds for tissue regeneration. In cartilage regeneration, they effectively encapsulate chondrocytes, enhance cell survival, reduce local inflammation, and facilitate joint cartilage repair [92]. For bone tissue engineering, RADA16 hydrogels loaded with basic fibroblast growth factor (bFGF) stimulate bone formation while protecting the growth factor from rapid degradation [119], addressing the critical challenge of preserving bioactive molecule stability in vivo.

Beyond native RADA16 hydrogels, functional modifications further expand their utility. Notably, RADA16 coupled with an antibacterial peptide (Amps) forms RA-Amps (retaining β-sheet assembly and antibacterial activity), which is embedded into PNIPAM thermosensitive hydrogels loaded with MGF E peptide (a fibroblast proliferation promoter) [120]. This composite (PNI/RA-Amps/E) undergoes phase transition at 32 °C and rapid reversible gelation (23 s), enabling injectable filling of irregular wounds. By integrating thermoresponsiveness, antibacterial activity, and sustained MGF E release, it accelerates wound healing in rat full-thickness wounds, outperforming commercial dressings as a clinical injectable candidate. Separately, thermosensitive chitosan hydrogels conjugated with recombinant human collagen-peptide (RHC) overcome the inherent limitations of pure chitosan hydrogels (weak mechanical strength and poor bioactivity) [121], further demonstrating the modular potential of peptide-modified hydrogel systems.

Second, aromatic short peptides (Phe, Tyr, and Trp) engage in π–π stacking interactions. Stacked aromatic rings confer stability for the encapsulation of hydrophobic drugs, forming hydrogels with architectures such as nanotubes, nanoribbons, or nanofibrils [122]. Representative examples include the dipeptide diphenylalanine (FF) and its derivatives (e.g., Fmoc-FF), which exhibit robust self-assembly [123]. Fmoc-FF is an ultrashort dipeptide gelator derived from the core sequence of β-amyloid protein. Its N-terminal fluorenylmethoxycarbonyl (Fmoc) group provides π–π stacking sites, while the C-terminal diphenylalanine (FF) mediates assembly through hydrophobic interactions and hydrogen bonds. With only two amino acid residues, Fmoc-FF can complete the entire process from molecular folding to gel network formation, featuring fast formation kinetics, easy accessibility, and mature technology. It has pioneered the paradigm for the study of “minimal peptide” self-assembly and stands as one of the most extensively investigated ultrashort peptides.

Notably, Fmoc-FF molecules exhibit two apparent pKa shifts (6.4 and 2.2), forming stable hydrogels in the pH range of 5.2–9.5, while assembly is inhibited at high pH (>10.5) due to ionization [124]. Common crosslinking methods to trigger assembly include solvent switching (e.g., HFIP/DMSO → water), pH adjustment (acid-induced protonation), temperature cycling (thermal dissolution and cold extraction), and changes in ionic strength [125].

However, it is worth noting that pure Fmoc-FF hydrogels possess a relatively low storage modulus, which cannot meet the mechanical requirements of load-bearing tissues (e.g., bone, cartilage) and thus require further enhancement through composite design. Additionally, the single functionality of hydrogels is insufficient to address complex clinical scenarios, necessitating the development of multifunctional delivery carriers. To address these challenges, various modification strategies have been developed based on the intrinsic tunability of peptides: cation-modified Fmoc-FFK to enhance antibacterial activity and gene delivery capacity [126]; Fmoc-FKFQF, Nap-FFY that modify specific sequences to achieve pH and salt response [126,127]; fluorinated Fmoc-3,4F-Phe, where single-atom substitution regulates assembly kinetics, improving mechanical stability and structural uniformity [128]; sequence extension with a photoresponsive group (tyrosine) to obtain Fmoc-FFY, thereby enhancing hydrophobic interactions and fluorescent properties [129]; and the introduction of cellulose nanofibrils (CNFs) to construct composite-modified uCNF/Fmoc-FF and CNF-g-Fmoc/Fmoc-FF systems, which significantly improve mechanical strength and 3D printability while optimizing the microscale network structure [130].

Many peptide hydrogels exhibit shear-thinning behavior and are injectable, transitioning to liquid states under stress and resolidifying at target sites to form in situ depots. Within this hydrogel network, drug molecules diffuse gradually as the gel degrades, enabling sustained release in tumor microenvironments. A NAP-modified Aβ fragment (FFKLVFF) serves as both a therapeutic agent and structural depot, self-assembling into an injectable gel with neuroprotective activity [131]. Peptide-based hydrogels are also particularly well-suited for constructing sustained-release drug-delivery systems. For instance, elastin-derived self-assembling peptides EDPs are designed based on natural elastin sequences—with Lys replaced by Asp/Glu to reduce positive charge and enhance network stability—self-assembling into hollow nanofibers in DMSO-Water systems. In skin anti-aging therapy, these EDP hydrogels achieve long-acting sustained transdermal delivery with pH-responsive release properties, outperforming free drugs [132]. Another representative example is the amphiphilic amino acid sequence-derived self-assembling hexamer peptide hydrogel, which loads asparaginase (Erwinase^®^) to develop an injectable sustained-release formulation for therapeutic enzymes. This system boasts excellent biocompatibility, prevents enzymatic degradation of the payload, and provides a universal platform for protein-based biologics, significantly improving clinical applicability [133]. Table 4 summarizes representative examples of peptide-based hydrogels and depots reported in recent years.

Nonetheless, several challenges persist for clinical translation. Key limitations include poor stability against physiological dilution and enzymatic degradation, difficulty in achieving predictive control and reproducible control over assembly morphology, low mechanical robustness, risk of burst drug release, and potential immunogenicity [134]. Strategies to address these challenges include chemical modifications (e.g., D-amino acids, cyclization), integration of stealth polymers, optimization of formulation conditions, and incorporation of stimuli-responsive network designs [135].

Overall, continued innovation in the design and engineering of self-assembling peptide materials will be essential to realize their potential for targeted delivery, tissue repair, and theranostics. Integrating multidisciplinary approaches—encompassing sequence bioengineering, advanced characterization techniques, and translational research—will further advance their impact in precision medicine.

## 4. Peptide-Based Linker for Targeted Therapy

**Figure 4:**
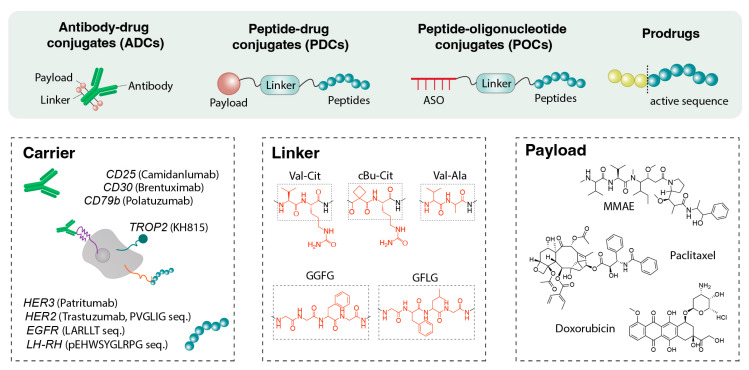
Components of peptide-based linkers. Antibody–drug conjugates (ADCs), peptide–drug conjugates (PDCs), peptide–oligonucleotide conjugates (POCs), and prodrugs utilize peptide-based linkers to connect therapeutic payloads to carriers such as antibodies against CD25, CD30, or CD79b, as well as targeting peptides directed toward HER2 or various growth factor receptors. These peptide linkers are engineered to provide both stability and controlled reactivity, enabling responsive cleavage under specific intracellular or pathological conditions for regulated payload release.

Conjugating drugs to targeting molecules via peptide linkers is an effective delivery strategy. Figure 4 demonstrates that in antibody–drug conjugates (ADCs), peptide–drug conjugates (PDCs), including peptide degrader (PROTACs/peptide-PROTACs), antisense oligonucleotide (ASO) conjugates, and siRNA conjugates, peptide linkers serve as biologically cleavable tethers that release the drug in response to specific stimuli (typically enzymes) at the target site. PDCs can be considered molecularly targeted delivery systems, essentially nanoscale vehicles in which the peptide functions as the targeting domain and the drug as the payload. The entire conjugate is typically 1–5 nm in size and behaves more like a small molecule than a nanoparticle, conferring distinct distribution properties. In ADCs, peptide linkers enable controlled intracellular release of cytotoxic payloads, usually via enzymatically cleavable sequences. These peptide linkers are designed to remain stable in plasma while being cleaved by tumor-associated proteases, such as cathepsins or legumain [136]. Recent efforts have focused on improving specificity and minimizing off-target activation by engineering sterically shielded or protease-selective peptide sequences to reduce systemic toxicity [137]. In PDCs, the linker connects a tumor-targeting peptide to a cytotoxic agent and is often optimized for enzymatic or pH-responsive cleavage [138]. The smaller size of PDCs offers enhanced tumor penetration compared with ADCs; however, it necessitates a careful balance between systemic stability and intracellular lability. Peptide-based linkers are also used in targeted radionuclide therapy. For example, Lutathera^®^, a peptide-radioisotope conjugate, comprises DOTA-TATE (a D-Phe^1^-Tyr^3^-octreotate peptide) that targets somatostatin receptors on neuroendocrine tumors and delivers the beta-emitting Lu-177 atom [139]. Although the payload is not a conventional-molecule drug, the principle remains the same: a peptide directs a toxic agent to disease tissue. Similar peptide-radioligand conjugates are in clinical trials for prostate cancer using PSMA-targeting peptides [140].

For ASO conjugates, peptide linkers facilitate both targeting and intracellular delivery. Linkers are typically engineered to respond to endosomal or cytosolic conditions, using disulfide bonds or enzyme-cleavable motifs to release the oligonucleotide cargo after cellular uptake [141]. Recent studies indicate that subtle changes in linker chemistry, such as the incorporation of self-immolative spacers, can substantially influence endosomal escape and gene silencing efficacy in vitro and in vivo [142]. Peptide-based linkers also enable tissue-specific activation of prodrugs. In this strategy, a cytotoxic peptide is rendered inactive by capping it with another peptide, which is selectively cleaved by an enzyme highly expressed in diseased tissue, resulting in localized activation of the therapeutic agent [143]. This section addresses peptide linkers in ADCs, PDCs and prodrug systems.

### 4.1. Peptide Linkers in ADCs

ADCs are complexes that link potent cytotoxic drugs to monoclonal antibodies that target tumor cells. The antibody and drug are connected via a chemical linker. The ideal peptide linker for an ADC is designed to remain intact and stable in the bloodstream, avoiding premature degradation, and to release the drug through enzymatic cleavage within or around the target cells. Proteases abundant in tumor cell lysosomes (e.g., cathepsins) are often overexpressed in tumors compared with normal tissue [144]. Peptide linkers responsive to these lysosomal proteases enable selective intracellular drug release. At the same time, these linkers remain relatively stable during blood circulation due to protease inhibitors in human plasma, thereby reducing premature drug leakage and minimizing systemic side effects.

For example, valine–citrulline (Val-Cit) is a dipeptide linker composed of two amino acids that is selectively cleaved by cathepsin B. This cleavage triggers a self-immolative breakdown, releasing the active drug inside the cell. Notably, the Val-Cit linker is stable in human plasma, ensuring that the drug remains attached to the antibody during circulation. Only internalized into a cancer cell, the acidic, enzyme-rich lysosomal environment cleaves the linker, releasing the drug to exert its cytotoxic effect [145]. Adcetris^®^ (brentuximab vedotin) employs a Val-Cit-PABC-MMAE linker-drug conjugate, which remains stable in blood and is cleaved in lymphoma cells to release MMAE, a microtubule toxin [146]. Polivy^®^ (polatuzumab vedotin) similarly utilizes a Val-Cit linker with MMAE for B-cell lymphoma, exhibiting stability in the bloodstream while releasing the drug upon lysosomal entry [147]. Overall, nine of the 14 currently approved ADCs incorporate peptide linkers such as Val-Cit. Additionally, many second-generation ADCs employ variations of this dipeptide linker or other peptide linkers, including Val-Ala or Gly-Gly-Phe-Met (GGFM) in trastuzumab deruxtecan [148]. The advantage of peptide linkers lies in their tunable stability and predictable enzymatic cleavage. Tetrapeptide linkers such as Gly-Phe-Leu-Gly (GFLG) or Gly-Gly-Phe-Gly (GGFG), a tetrapeptide linker composed of four amino acids, are cleaved by proteases, including cathepsins, within the tumor microenvironment or intracellular organelles. For example, the third-generation ADC trastuzumab deruxtecan (Enhertu™) uses a GGFG-based linker to connect a topoisomerase inhibitor. This linker stably delivers the drug into the bloodstream and is enzymatically degraded by lysosomal enzymes such as cathepsin L, upon reaching tumor tissue, releasing the active drug [149]. These mechanisms enable Enhertu™ to demonstrate therapeutic efficacy even in cancer cells with low human epidermal growth factor receptor 2 (HER2) expression [150,151].

Peptide linkers in ADCs are predominantly developed for oncology applications, with enzyme-cleavable designs being the most common. Chemical cleavable linkers, such as pH-sensitive hydrazone bonds or reducible disulfide bonds, have also been utilized in ADCs; however, peptide linkers are preferred due to their high stability and selective responsiveness to tumor-associated enzymes [152]. In contrast, non-cleavable linkers permanently bind the drug to the antibody, releasing it only upon complete antibody degradation within tumor cells, which generally results in lower cytotoxic efficacy. Therefore, peptide linkers that undergo enzymatic cleavage, such as Val-Cit or GGFG, are preferred when rapid and efficient drug release at the target site is required.

Some ADCs utilize peptide linkers responsive to alternative triggers, such as metalloproteinase-cleavable sequences that release the drug in the tumor microenvironment rather than intracellularly. For example, a peptide sequence recognized by matrix metalloproteinases 2 and 9 (MMP-2/9), such as PLGLAG, can be cleaved by extracellular MMPs in invasive tumors, thereby activating the conjugate within the tumor microenvironment [153]. Table 5 summarizes representative peptide linkers used in ADCs, along with their cleavage mechanisms, functional roles, and amino acid sequences.

### 4.2. Peptide Linkers in PDCs

PDCs are a next-generation targeted therapy platform that functions as “mini-ADCs,” directly linking target-specific peptides to small-molecule drugs. While structurally similar to ADCs, PDCs use small peptides as carriers rather than antibodies, offering advantages such as ease of manufacturing, high tissue penetration due to their low molecular weight, rapid clearance from the body, and reduced immunogenicity. Peptides are gaining attention as cost-effective, flexible drug-delivery vehicles because they are easily synthesized or chemically modified (e.g., cyclization or incorporation of non-natural amino acids), and even short sequences comprising a few dozen amino acids can achieve sufficient target recognition [154]. These advantages have facilitated the development of PDC as a therapeutic candidate for a variety of diseases, including cancer, metabolic disorders, and viral infections (e.g., COVID-19) [155,156].

A PDC generally consists of a targeting peptide (or cell-penetrating peptide), a linker, and a drug payload. The design principle is similar to that of ADCs: the peptide binds to specific receptors or antigens at the disease site, delivering the drug to the target area, while the linker responds to the environmental cues to release the drug. Peptide ligands can be designed to target receptors overexpressed in tumor tissue (e.g., luteinizing hormone–releasing hormone (LHRH) receptor, integrin α_v_β_3_, epidermal growth factor receptor [EGFR]), angiogenesis markers, or BBB receptors [157]. Notably, advanced homing peptide designs have expanded targeting precision and barrier-penetrating capabilities: Angiopep2, which binds to low-density lipoprotein receptor-related protein 1 (LRP1), enables BBB crossing when conjugated with engineered extracellular vesicles for brain-targeted drug delivery [158]; a rationally designed “Y-shaped” DWVAP homing peptide, constructed by linking DVAP (targeting GRP78 protein) and the BBB-penetrating quorum-sensing peptide DWSW via alanine, exhibits exceptional tropism for glioma cells (including glioma stem cells, GSCs) and enhanced tumor targeting through dual functional integration [159]; PL3, a Tenascin-C-targeting peptide, further strengthens delivery efficacy by interacting with the cell/tissue-penetrating receptor neuropilin-1 (NRP-1) via its C-terminal CendR motif, holding promise as a versatile scaffold for targeted delivery of imaging agents and therapeutics to solid tumors [160]. Beyond tumor-targeting, homing peptides also extend to non-oncological diseases: SHp, a peptide isolated via in vivo phage library screening, shows high affinity for glutamate receptors in oxidatively stressed apoptotic neurons, and its modification enables accelerated targeted drug delivery to damaged neurons in stroke lesions [161].

The linker and drug-release mechanism often employ chemically labile bonds that degrade in response to environmental conditions, similar to those in ADCs. Because peptides are relatively small molecules, direct single-bond attachment of the drug to the peptide is also common, although enzyme- or pH-sensitive peptide spacers can be incorporated when necessary. For example, a PDC linking a HER2-targeting peptide to doxorubicin (DOX) via the six-amino acid sequence PVGLIG, which is cleaved by MMP-2, has been reported [162]. Cleavage by MMP-2 in the tumor microenvironment activates the drug, resulting in potent anticancer effects and prolonged circulation half-life. In addition to enzyme-cleavable linkers, acid-sensitive linkers have been employed in PDC. For example, DOX conjugated to peptides via hydrazone bond hydrolyzes under acidic conditions (pH 5–6), releasing the drug within the low-pH tumor environment [163]. However, hydrazone linkers exhibit limited plasma stability, prompting the development of more stable alternatives. Succinic acid-based ester linkers, connecting hydrophilic cell-penetrating peptide (e.g., c[RGDKLAK]) to paclitaxel, improve water solubility and enable controlled drug release under acidic conditions, demonstrating substantial tumor-growth inhibition in mouse glioblastoma models [164]. Furthermore, disulfide bonds responsive to intracellular reducing environments are also utilized. In tumor tissue, intracellular concentrations of the reducing agent glutathione (GSH) are more than four times higher than in normal tissue. Incorporating disulfide linkers allows selective drug release within the reducing environment of cancer cells [165]. Cyclic RGD peptides conjugated to the DM1 toxin via disulfide bonds have demonstrated tumor-suppressive effects while exhibiting rapid renal excretion and low hepatic accumulation, highlighting their clinical potential [166]. Table 6 summarizes representative peptide linkers used in peptide–drug conjugates (PDCs), along with their responsive mechanisms and key drug delivery characteristics.

The therapeutic success of ADCs and PDCs depends on a delicate balance: the linker must be sufficiently stable in circulation to prevent premature drug release and off-target toxicity, yet labile enough to release the active drug within the target environment. Peptide linkers provide a biologically compatible approach to achieve this balance. By employing sequences cleaved by enzymes predominantly present at the disease site, high specificity can be attained. In cancer targeting PDCs, where payloads are highly potent cytotoxins, precise linker cleavage is critical to minimize systemic toxicity. Peptide linkers can also mitigate bystander effects by controlling the diffusion of released drugs. Thus, linker design integrates considerations of enzyme specificity, peptide stability, circulation time, and desired drug-release kinetics.

### 4.3. Peptide-Based Prodrugs

A prodrug is an inactive precursor that is converted into an active drug by specific biochemical conditions or enzymes in the body. Peptide-based prodrugs attach short peptide sequences to existing drugs to transiently mask activity or to target delivery. An appropriate peptide “mask” can reduce systemic toxicity during circulation and be cleaved at the target site to release the active drug. Peptide sequences are designed to be selectively cleaved by enzymes that are upregulated or activated in diseased tissues. Mipsagargin (G-202) is an example of a peptide-masked prodrug. It comprises a thapsigargin derivative linked to a prostate-specific membrane antigen (PSMA) recognition peptide [167]. In normal tissue, the conjugate remains largely inactive, but in PSMA-rich tumor vasculature and tumor cells, the peptide mask is cleaved by PSMA, releasing the active thapsigargin. Thapsigargin irreversibly inhibits the sarcoplasmic/endoplasmic reticulum calcium ATPase (SERCA), inducing cell death [168]. Mipsagargin demonstrated a favorable safety and pharmacological profile in early clinical trials, and clinical development has continued across several solid tumor indications, including hepatocellular carcinoma and brain tumors [169].

Peptide-based prodrugs that utilize aminopeptidases or endopeptidases are gaining considerable attention in oncology. Melphalan flufenamide (Melflufen) is a peptide prodrug developed for the treatment of multiple myeloma. It consists of the alkylating agent melphalan conjugated to a hydrophobic dipeptide, which enhances cell membrane permeability compared with the parent compound, facilitating efficient uptake into cancer cells. Inside the cells, the dipeptide portion is rapidly cleaved by overexpressed aminopeptidases, releasing the active form of melphalan. Consequently, Melflufen demonstrates 10- to 100-fold greater cytotoxicity than melphalan [170].

The following peptide sequences and cleavage mechanisms are commonly employed in peptide-based prodrugs. The tripeptide sequence Ala-Ala-Asn (AAN) is specifically cleaved by the cysteine protease legumain. Because legumain is frequently overexpressed in tumor cells and tumor microenvironment macrophages, AAN linkers enable preferential activation within the tumor microenvironment, thereby reducing toxicity to normal tissues [171]. However, short peptide linkers such as AAN may exhibit limited stability in the bloodstream, resulting in premature drug release during preclinical evaluations. Hydrophobic dipeptides, typically composed of two to three amino acids such as Phe or Leu, offer advantages, including enhanced drug membrane permeability and susceptibility to cleavage by specific aminopeptidases. As demonstrated with melphalan flufenamide, conjugation of hydrophobic dipeptides to alkylating agents or cytotoxic compounds can facilitate rapid intracellular drug release in cancer cells that overexpress the corresponding enzyme [172]. Similarly, peptide precursors cleavable by tumor- or tissue-specific enzymes, such as prostate-specific antigen (PSA) or MMP-activating sequences, are being explored to develop prodrugs targeted to prostate cancer or the tumor microenvironment.

Peptide-based prodrug strategies can be applied not only in oncology but also in the treatment of inflammatory diseases and in the delivery of central nervous system drugs. For example, in chronic inflammation such as arthritis, specific MMP family enzymes are highly expressed, enabling the development of anti-inflammatory prodrugs that are selectively activated at sites of inflammation via MMP-cleavable peptide linkers [173]. In CNS applications, drugs can be conjugated to amino acid-mimicking peptides to facilitate passive transport across the BBB via amino acid transporters in cerebral blood vessels, followed by enzymatic activation within brain tissue [174]. Overall, prodrug design that leverages the biological specificity and enzymatic targeting of peptides is emerging as a powerful approach to overcome the limitations of conventional drugs and improve the precision of drug delivery. Strategies aimed at maximizing efficacy and safety, including multifunctional peptide linkers with dual targeting and self-assembly capabilities or advanced drug-peptide conjugation chemistry, are increasingly prominent. These developments are expected to enhance the precision of targeted therapies and reduce side effects across various medical fields, including oncology, chronic inflammatory disorders, and neurodegenerative diseases.

## 5. Sequence-Based Design Strategies for Optimizing Peptide Delivery Systems

**Figure 5:**
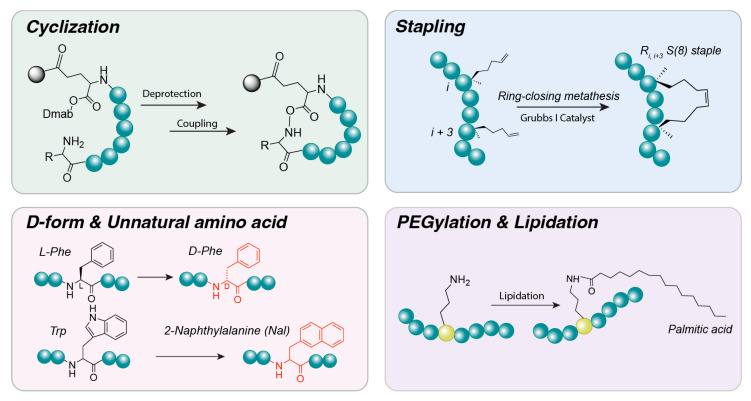
Peptide design and optimization. Various strategies can be employed to modify peptide sequences and enhance their structural or functional properties. Cyclization can be achieved through the reaction of Dmab-terminal amines, while stapled peptides can be generated using ring-closing metathesis. Additional modifications include substitution of L-amino acids with D-amino acids or unnatural amino acids to improve stability, as well as lipidation and PEGylation through conjugation of fatty acids or polyethylene glycol (PEG), respectively.

Design strategies at the amino acid sequence level are being actively employed to enhance the performance of peptide delivery systems. The objective is to manipulate the primary sequence of peptides to improve stability, cell permeability, targeting specificity, and other functional properties. This section explores sequence-based design strategies from three perspectives: rational design and biophysical optimization, the introduction of functional motifs, and the utilization of combinatorial libraries and artificial intelligence (AI). As illustrated in Figure 5 a key advantage of peptides lies in their tunable amino acid sequences, which can be engineered to achieve desired functions. Rational design, motif incorporation, and combinatorial selection are employed to develop peptide biomaterials with enhanced delivery, targeting, and stability.

### 5.1. Rational Design and Biophysical Optimization

Various chemical and physical optimization techniques are applied to enhance the structural stability and in vivo persistence of peptides. These approaches aim to overcome intrinsic weaknesses of peptide sequences, such as susceptibility to proteolytic degradation and structural flexibility, by modifying the peptide sequence or introducing specialized bonds. Key techniques include:

*Cyclization:* Formation of cyclic structures through covalent bonds between the N- and C-termini or between side chains of peptide molecules. Strategies include head-to-tail coupling and side-chain bridge formation [175]. Cyclization has been shown to substantially increase proteolytic stability relative to linear peptides and, in some cases, enhance cell membrane permeability [176]. By constraining the peptide backbone into rigid turns or loops, cyclization can pre-form secondary structures, such as β-turns, and stabilize α-helical or β-sheet motifs [177]. These structural constraints help lock the peptide into physiologically active conformations that are favorable for target binding. Cyclizing a peptide (head-to-tail or via a disulfide bridge) often increases its stability against proteases and locks it into a bioactive conformation. For example, octreotide, a cyclic somatostatin analog, exhibits marked stability and potency. Similarly, cyclic RGD peptides (e.g., cRGDfK) exhibit higher affinity and greater resistance to degradation, rendering them suitable for targeting tumors [178].

*Stapling and helical stabilization:* Short peptides often struggle to maintain an alpha-helical (α-helix) structure in solution. Helical stabilization can be achieved by introducing crosslinks between side chains, such as lactam bridges (amide bonds between glutamic acid and lysine) or disulfide bonds between amino acids at positions *i*, *i + 4*, or *i + 7* [179]. Hydrocarbon stapling techniques have also been developed, in which amino acids are replaced with non-natural residues to form hydrocarbon rings. These “staple” bonds enforce the α-helical conformation, reducing susceptibility to proteolytic degradation and enhancing utility in applications such as protein–protein interaction inhibitors [180]. Additional strategies aim to form helical structures by replacing intramolecular hydrogen bonds with covalent bonds, as exemplified by the hydrogen bond surrogate technique [181]. Collectively, these cross-links enable even small peptides to adopt rigid secondary structures, thereby increasing in vivo stability and target-binding affinity. “Stapled peptides” are peptides constrained into an α-helix through an artificial cross-link, typically a hydrocarbon staple between *i* and *i + 4* residues. This approach improves cell permeability and protease resistance and is particularly useful for peptides targeting intracellular protein interactions. Examples include ALRN-6924, a stapled α-helix of p53 that penetrates cells to inhibit MDM2 and demonstrates efficacy in leukemia models [182], and stapled glucagon-like peptide-2 (GLP-2) analogs, which exhibit prolonged activity for the treatment of intestinal disorders [183].

*D-amino acids and unnatural residues:* One of the primary limitations of peptides is their susceptibility to proteolytic degradation. To address this challenge, non-natural amino acids are often incorporated into peptide sequences. Substituting *L*-amino acids with their mirror-image D-amino acids renders them unrecognizable to peptide hydrolases, markedly enhancing resistance to enzymatic degradation. Several clinically essential peptide drugs are composed entirely or partially of D-amino acids [184]. Additionally, incorporation of beta-amino acids or peptoids (N-substituted glycines) in place of alpha-amino acids modifies the peptide backbone, further reducing protease recognition. N-methylation of amino acids is another strategy that impedes protease access and increases cell membrane permeability by replacing the hydrogen atom in the peptide bond with a methyl group [185]. Although D-amino acid substitution provides pharmacokinetic advantages, such as extended half-life, it may alter interactions with the original target, potentially reducing activity [186]. Overall, incorporation of D-amino acids or other non-natural residues can substantially slow peptide breakdown in vivo. For example, desmopressin, a vasopressin analog used to treat diabetes insipidus, contains a D-arginine residue at the C-terminus, which confers resistance to carboxypeptidases and prolongs its activity. Similarly, experimental peptide drugs frequently employ D-amino acid substitution or N-methylation of amide bonds to evade proteolysis [187].

*PEGylation and lipidation:* These strategies are widely used to improve the pharmacological properties of peptide therapeutics by attaching polymer chains or lipid moieties to the peptide structure. PEGylation refers to the covalent attachment of polyethylene glycol (PEG) chains, which increases the molecular weight and hydrodynamic size of peptides, thereby reducing filtration and prolonging systemic circulation time. PEG is highly biocompatible and exhibits low immunogenicity, and has been used since the 1970s to extend the half-life of protein and peptide drugs. Numerous PEGlyated therapeutics are currently approved for clinical use. PEGylation also enhances peptide solubility and stability by providing shielding from proteolytic enzymes [188]. Lipidation involves covalently attaching fatty acid chains to peptides, commonly at a Lys residue or the N-terminus. A well-established example is the GLP-1 analogs liraglutide and semaglutide, which incorporate C16- or C18-fatty acid chains, respectively [189]. These lipid moieties increase albumin binding affinity and markedly extend plasma half-life. Insulin degludec also utilizes fatty acids to facilitate reversible albumin binding and prolong its circulation time [190]. Although PEGylation and lipidation chains do not alter the peptide sequence itself, the peptide backbone is often modified to incorporate an appropriate attachment site, frequently by inserting a short spacer such as a dipeptide. Pegvisomant, a growth hormone receptor antagonist used in the treatment of acromegaly, is an example of a clinically approved PEGylated peptide analog [191].

### 5.2. Motif Incorporation for Targeting and Function

A widely used strategy for precise delivery of peptides to specific cells or organelles is the incorporation of functional motifs into the peptide sequence. Although short, these motifs enhance delivery efficiency and specificity by providing biological functions such as receptor targeting, membrane penetration, and organelle localization.

*Targeting ligands:* Peptides can be directed to specific short tissues or cells by attaching short ligand sequences that bind to target receptors. A well-studied example is the RGD motif (Arg-Gly-Asp), which exhibits high affinity for integrin receptors (α_v_β_3_, α_v_β_5_) and is therefore employed to selectively deliver peptides to tumor neovasculature or cancer cells [192]. RGD-functionalized nanoparticles have demonstrated increased accumulation in tumor tissue with reduced distribution in normal tissue, thereby enhancing specificity and therapeutic efficacy. The NGR motif (Asn-Gly-Arg selectively binds to aminopeptidase N (CD13) and accumulates in tumor vasculature. These targeting motifs are often identified through combinatorial screening techniques such as phage display, and when incorporated into drug delivery systems, confer active targeting to the desired tissue [193]. Short peptide motifs that bind receptors or tissues can be fused to therapeutics or carriers to achieve targeted delivery. For example, RGD and its cyclic variants bind integrin receptors overexpressed on tumor endothelium. Bombesin/gastrin-releasing peptide (GRP) motifs have been conjugated to radiopharmaceutical peptides to target GRP receptors in prostate and breast cancers. Additionally, peptide motifs can mimic monoclonal antibody epitopes to engage the same receptor, enhancing the specificity and versatility of targeted therapies [194].

*Cell-penetrating sequences:* Amphipathic or cationic cell-penetrating peptides facilitate intracellular delivery of therapeutics that cannot efficiently cross cell membranes on their own. A canonical example is the TAT peptide (TAT_48–60_), derived from HIV-1, an 11-residue polycationic sequence that efficiently translocates into diverse cell types [195]. Other CPPs include penetratin and polyarginine sequences [196]. CPPs are typically 5–30 amino acids in length and interact with cell membranes via positive charge residues (Lys, Arg) or a hydrophobic and amphipathic motif Chemical conjugation of CPPs to peptide drugs, nucleic acids, and nanoparticles enables efficient internalization via energy-dependent endocytosis and other pathways [197]. However, CPP-cargo complexes can be sequestered in endosomes after internalization; consequently, endosomal escape motifs are often co-incorporated. For instance, polyarginine sequences (e.g., R9) or TAT (YGRKKRRQRRR) can be appended to cargo peptides [22,198]. Many research-stage therapeutic targeting intracellular pathways consist of the active peptide fused directly to a CPP. For example, a 20-mer inhibitor of JNK signaling was rendered cell-permeable by a polyarginine tail, enabling effective treatment of inflammation in murine models [199].

*Nuclear localization signals or mitochondrial targeting sequences:* To deliver a drug beyond the cytoplasm to a specific organelle, peptides can be appended with organelle-specific targeting signals. Nuclear localization signals (NLS) are short sequences composed of basic amino acids that are recognized by nuclear import receptors, such as importins, facilitating transport through nuclear pores. For example, the SV40 large T antigen NLS (Pro-Lys-Lys-Lys-Arg-Lys-Val) can be attached to proteins or nanoparticles to promote nuclear entry [200]. Nanoparticles incorporating NLS peptides have demonstrated nuclear delivery efficiencies several-fold higher than particles lacking this motif. Mitochondrial targeting sequences (MTS) are typically 20–40 amino acid amphipathic α-helical peptides appended to the N-terminus of proteins synthesized in the cytoplasm. These sequences are recognized by receptors on the mitochondrial outer membrane and mediate transport into the organelle. Linking an MTS to a peptide carrier can target the carrier or its therapeutic cargo to mitochondria. Experimental strategies have delivered therapeutic enzymes to mitochondria by fusing MTS sequences to cell-penetrating peptides such as TAT [201,202]. Incorporation of organelle-specific motifs maximizes the therapeutic effect at the desired intracellular location. For nuclear targeting, sequences such as KKKRKV can be employed, whereas mitochondrial targeting peptides typically contain Arg, Ser, and Thr residues with amphipathic helical propensity. Although largely experimental, these approaches illustrate how modular peptide design can achieve subcellular localization.

*Hydrophobic or β-sheet motifs for assembly:* Self-assembly of peptides into nanostructures can be driven by hydrophobic patterns or β-sheet-forming sequences complementary to the peptide backbone. Ionic complementary peptides, such as RADA16-I (Ac-(RADA)_4_-CONH_2_) [119] and EAK-16 [203], feature alternating positively charged Arg and negatively charged Asp residues, which enable electrostatic interactions and formation of stable β-sheet nanofibrils. In acidic solutions, they remains soluble as individual peptides, but upon exposure to neutral physiological pH, it rapidly self-assembles into β-sheet nanofibers, which intertwine to form a transparent hydrogel matrix.

Beyond naturally derived or ionic complementary sequences, rationally designed β-hairpin peptides like MAX1 (VKVKVKVKVDPPTKVKVKVKV-NH_2_) [204] and its derivative MAX8 (with K15E substitution) [205] represent paradigmatic examples of β-sheet-driven assembly. These peptides harbor amphiphilic β-strands connected by a V^d^PPT type II’ β-turn, and their self-assembly is triggered by physiological ionic strength (e.g., 150 mM NaCl) or cell culture media (e.g., DMEM), which shields electrostatic repulsion between lysine residues to promote β-hairpin folding and subsequent antiparallel β-sheet stacking. Solid-state NMR and molecular dynamics (MD) simulations confirm that MAX peptides form stable cross-β nanostructures with a core of hydrogen-bonded β-sheets and hydrophobic valine-rich faces, yielding ~3 nm-wide nanofibers that further cross-link into hydrogels. Notably, MAX8 exhibits accelerated β-sheet formation (from 30 min to <1 min) due to reduced net charge, and its hydrogel retains shear-thinning and self-healing properties, making it suitable for cell encapsulation and injectable tissue engineering scaffolds.

These nanostructures are sufficiently stable for use as scaffolds in tissue engineering, and can serve as sustained-release systems when therapeutic agents are encapsulated within the hydrogel. Peptide nanoparticles, micelles, and capsules have also been constructed using β-sheet motifs, such as KLVFF from the amyloid-β (Aβ) sequence, or other flat hydrophobic sequences [206]. The inclusion of self-assembly motifs enables peptide carriers to form stable nanostructures, enhancing in vivo stability and enabling multifunctional delivery. For example, repeating Ala or Val can promote β-sheet formation, whereas FF motifs nucleate nanotube assembly. The FF dipeptide core represents a minimal self-assembling unit that stacks into nanotubes, which have been extensively investigated for drug delivery and nanotechnology applications [207,208]. Incorporation of such motifs into peptide sequences enables controlled aggregation into desired nanostructures. Table 7 presents the motif incorporation for targeting and function.

### 5.3. Combinatorial Discovery Using Phage Display, Diverse Libraries, and AI Assistance

In peptide design, it is challenging to anticipate all possible sequence-function scenarios through rational design alone. Consequently, approaches that utilize large-scale combinatorial libraries, often combined with AI techniques, are increasingly employed to identify optimal peptide sequences [209]. Combinatorial methods enable the discovery of peptide with desired properties without requiring prior assumptions about sequence-function relationships.

*Phage display:* A powerful technique for identifying peptides that bind to specific targets, including receptors, enzymes, and tissues. Large libraries containing approximately 10^9^ bacteriophage variants, each displaying a random peptide, can be panned against a target to enrich for high-affinity binding sequences. This approach has led to the discovery of numerous targeting peptides. For example, Angiopep-2, which facilitates transport across the BBB, was identified via a phage display screen targeting endothelial cells [210]. Similarly, iRGD, a tumor penetrating peptide, and LyP-1, a tumor-homing peptide for lymphatics, were identified using in vivo phage display in tumor-bearing mice [211,212]. Phage display has also been employed to identify peptide substrates for enzymes and to discover or optimize CPPs [213].

*Random peptide library screening:* Enables the selection of sequences that fulfill a specific function from a diverse pool of peptides. Traditionally, this approach relies on phage display, in which billions of random peptide sequences are expressed on the coat proteins of bacteriophages. Selective biopanning isolates phages that bind to or enter specific cells or receptors. This method has successfully identified peptides that home to blood vessels in target organs, penetrate specific cell types, or perform other desired functions. For example, phage display has facilitated the discovery of peptides capable of crossing the BBB or penetrating tumor tissue, which can subsequently be attached to nanodrug delivery systems [214]. In vivo phage display techniques have further enabled the selection of phages that specifically of tissue-specific peptides within a biological environment, yielding ligands targeting various organs. In addition, combinatorial library approaches have also been applied to identify CPPs for intracellular delivery. An illustrative example is the NNJA phage display platform, which discovered novel CPP sequences efficiently delivered to the cytoplasm using a library designed so that phages replicate only after endosomal escape [213]. Overall, combinatorial discovery techniques enable the identification of non-intuitive sequences that are difficult to predict, uncovering candidates with high potential for targeted peptide delivery systems.

*AI and computational design:* With advances in AI, machine learning, and deep learning, these techniques are increasingly employed in peptide sequence design. Traditionally, peptide drug development relied on experimentation and empirical knowledge, necessitating the testing of large numbers of candidate sequences individually. However, AI can leverage extensive chemical and biological datasets to predict sequences with desired properties, such as high target affinity and resistance to degradation. For example, Evolutionary Scale Modeling (ESM) can generate and evaluate millions of peptide sequences, identifying candidates that strongly bind specific receptors while remaining stable [215]. Companies such as Nuritas have developed platforms that analyze databases comprising hundreds of millions of protein sequences using deep learning, predicting bioactive peptide fragments that are li subsequently experimentally validated. AI, combined with 3D structural prediction, also enables the design of peptides that co-optimize structural complementarity with their targets. Furthermore, generative models, evolutionary algorithms and reinforcement learning are being explored to accelerate sequence generation and optimization [216]. Recent studies indicate that AI-generated peptide include combinations difficult to predict using traditional design methods and can exhibit high experimental activity. Advances in computational biology have also enabled the design peptide sequences for specific structural or functional requirements [217]. A striking example is the super learning-based design of multifunctional peptide HR97—engineered for melanin binding, cellular internalization, and low cytotoxicity—which directly addresses poor adherence to frequent dosing in chronic ocular diseases. Conjugated to thrice-daily dosed brimonidine, HR97 forms an ocular sustained-release depot via melanin binding, achieving 18-day efficacy [218].

AI can optimize sequences for stability, binding affinity, or self-assembly by learning from large datasets. For instance, computational modeling of peptide-drug interactions in nanoparticle systems has narrowed the candidate selection for experimental testing [219,220]. Computational protein design tools, initially developed for protein folding, are now being adapted for the de novo design of self-assembling peptides and peptide carriers with controlled release profiles. For example, algorithms can design peptides that form a cage-like nanoparticle of defined dimensions, which degrades selectively in the presence of specific proteases [221].

In summary, sequence-based strategies provide precise control over peptide biomaterials. By modifying amino acid composition or incorporating functional motifs, peptides can be tailored to enhance targeting, uptake, stability, circulation time, and controlled release of payloads. The integration of rational design with combinatorial discovery has substantially expanded the repertoire of functional peptides for drug delivery applications, facilitating both the optimization of known systems and the exploration of novel solutions.

## 6. Conclusions and Future Perspectives

Peptide biomaterials for drug delivery represent a rapidly advancing field at the intersection of chemistry, biology, and engineering. Therapeutic peptides have already demonstrated substantial clinical value, and emerging delivery modalities, including oral peptide formulations and peptide-targeted nanomedicines, are expanding their therapeutic scope. Peptide-based excipients and linkers also facilitate site-specific drug action, addressing the longstanding challenge of selectively delivering to diseased tissues while minimizing effects on healthy cells. The mechanisms by which peptides function, such as receptor binding, membrane penetration, self-assembly, and enzymatic responsiveness, are closely aligned with intrinsic biological processes. As a result, peptide-based biomaterials are typically biocompatible and biodegradable into native amino acids. Peptide-based systems have therefore progressed from a niche concept to a central strategy in drug delivery and pharmaceutical development, with multiple examples already in clinical use and a substantial pipeline of candidates in various stages of evaluation. By leveraging the unique mechanisms of action of peptides, whether as therapeutics, delivery agents, or responsive linkers, researchers can design treatments that are more targeted, effective, and safe. As computational, synthetic, and combinatorial peptide design methodologies continue to advance, the potential for innovative peptide-based biomaterials to address unmet medical needs is expected to grow accordingly.

## Figures and Tables

**Table 1:** Representative examples of CPPs along with their corresponding sequences.

Category	Peptide Name	Sequence	Origin	Ref.
Cationic				
	TAT	GRKKRRQRRRPQ	HIV-1 protein	[17]
	R9	RRRRRRRRR	Synthetic	[22]
	R12	RRRRRRRRRRRR	Synthetic	[25]
	pVec	LLIILRRRRRKQAHAHSK	Cadherin protein	[24]
	P22N	RRRQRKRGY	Phage P22 capsid protein	[26]
	RTP004	RKKRRQRRR	Synthetic	[21]
	Penetratin	RQIKIWFQNRRMKWKK-NH_2_	Antennapedia protein	[47]
Amphipathic				
	MPG	GALFLGFLGAAGSTMGAWSQPKKKRKV	HIV-1 gp41	[28]
	Pep-1	KETWWETWTEWSQPKKKRKV	Synthetic	[29]
	MAP	KLALKLALKALKAALKL	Synthetic	[31]
	TP10	AGYLLGKINLKALAALAKKIL	Galanin and mastoparan	[48]
	Transportan	GWTLNSAGYLLGKINLKALAALAKKI	Galanin and mastoparan	[37]
	SAP	VRLPPPVRLPPPVRLPPP	*γ*-zein	[38]
Hydrophobic				
	Hel13-5	KLLKLLLKLWLKLLKLLL	Human pulmonary surfactant protein B	[41]
	PFVYLI	PFVYLI	Synthetic	[42]
	Mastoparan	INLKALAALAKKIL	Hymenopteran venom	[49]
	Pept-1 (TP1)	PLILLRLLRGQF	Synthetic	[44]
	Pept-2 (TP2)	PLIYLRLLRGQF	Synthetic	[44]
	Pep-7	SDLWEMMMVSLACQY	HIV-1 protein	[45]
	IVV-14	KLWMRWYSPTTRRYG	Synthetic	[46]

**Table 2:** Representative examples of peptide-based nanospheres.

Peptide Name	Sequence	Function	Ref.
L,L-cyclic	WRWRWRWRCR WWWWGGRRRRGC WRWRCRWRCR	Exhibit strong binding affinity toward siRNAs, Antisense oligonucleotides	[86]
EOG-10	G(EOG)_4_EOG(EOG)_5_	TPE-PRG forms nanospheres via EOG-10 mediation, exhibiting excellent luminescence properties and potential for fluorescent tracing.	[87]
RGD motif-containing peptide	VVVVVKKGRGDS	pH-responsive and tumor-targeted drug delivery	[89]
tLyP-1	CGNKRTR	NRP-1-targeted tumor-specific tissue penetration	[92]
Cyclic peptide	(WR)_5_C	GdCl_3_-integrated theragnostic platform for MRI-tracked drug delivery	[93]
PEG6-Y4	H_2_N-PEG_6_-Y-Y-Y-Y	Transforms into fluorescent nanospheres upon heating, serving as a bio-diagnostic tool.	[94]

**Table 3:** Representative examples of nanovesicles and micelles.

Peptide Name	Sequence	Function	Ref.
A6D V6K	AAAAAD VVVVVK	Mimicking lipid architectures, hydrophobic core can encapsulate poorly soluble drugs, enhancing solubility, stability, and biocompatibility.	[106]
GSLP	GNNQQNY-PKKP-GNNQQNY	Self-assembles into nanocages, enabling high drug loading and enhanced solubilization.	[111]
APK	AAAAAAPKKPAAAAAA	Capable of efficiently encapsulating hydrophobic drugs while exhibiting intrinsic antimicrobial activity.	[113]
RD2	PTLHTHNRRRRRR	Exhibits high drug-loading capacity and selectively inhibits amyloid-β aggregation in Aβ	[115]

**Table 4:** Representative examples of peptide-based hydrogels and depots.

Peptide Name	Sequence	Function	Ref.
EAK16	AEAEAKAKAEAEAAKAK	Self-assembles into stable nanoscaffolds in water.	[117]
RADA16-I	RADARADARADARADA	Self-assembles into an ECM nanohydrogel that protects encapsulated APIs while supporting 3D cell culture and promoting bone regeneration.	[118]
RHC	(G-P-P-G-E-K-G-P-A)_n_	Conjugation with chitosan yields a thermosensitive hydrogel with enhanced mechanical strength and improved bioactivity.	[121]
Fmoc-FF	Fmoc-FF	As a paradigm in minimalistic peptide self-assembly.	[123]
Fmoc-FFK	Fmoc-FFK	Cationic modification enhances both antimicrobial activity and delivery efficiency.	[127]
Fmoc-FKFQF	Fmoc-FKFQF	PH and salt-responsive	[127]
Nap-FFY	Nap-FFY	In addition to pH and salt-responsiveness, it exhibits enhanced hydrophobic interactions and intrinsic fluorescence.	[129]
Fmoc-3,4-difluorophenylalanine	Fmoc-3,4F-Phe	Fluorination enhances mechanical stability and structural homogeneity.	[128]
EDP-1 EDP-5	Fmoc-FFAAAADAA-NH Fmoc-FFAAAAAAA-NH_2_	Enables sustained transdermal delivery with pH-responsive release.	[132]
Hexamer peptide	H-FEFQFK-OH	Peptide acts as a gelator to form an injectable, sustained-release hydrogel with excellent biocompatibility and resistance to enzymatic degradation.	[133]
NAP-modified Aβ fragment	NAP-FFKLVFF	Functioning as both a therapeutic agent and a structural reservoir, it self-assembles into an injectable hydrogel with neuroprotective activity.	[131]

**Table 5:** Representative examples of Peptide linkers in ADCs.

Peptide Name	Sequence	Function	Ref.
Val-Cit	V-Cit	Selectively cleaved by cathepsin B in tumor cell lysosomes to release the APIs.	[145]
Val-Ala	VA	Enzymatically cleavable for drug release, stable in plasma, and suitable for tumor-targeted delivery.	[148]
Gly-Gly-Phe-Met	GGFM	Enables enzyme-triggered drug release with high systemic stability and tumor-targeting capability.	[148]
Gly-Phe-Leu-Gly	GFLG	Exhibits high drug-loading capacity and selectively inhibits amyloid-β aggregation in Aβ	[149]
Gly-Gly-Phe-Gly	GGFG	Exhibit high stability in plasma, is selectively cleaved by lysosomal cathepsin L, and releases the APIs—retaining therapeutic efficacy even against cancer cells with low HER2 expression.	[150]
PLGLAG	PLGLAG	Cleaved by MMP-2/9 in the extracellular microenvironment of invasive tumors to release the drug.	[153]

**Table 6:** Representative examples of Peptide linkers in PDCs.

Peptide Name	Sequence	Function	Ref.
Angiopep2	TFFYGGSRGKRNNFKTEEY	Targeting LRP1 and conjugated to EVs can crosses the BBB for brain-targeted drug delivery.	[158]
DWVAP	DVAP-Y-DWSW	A bifunctional integrated design confers high glioma tropism and enhances tumor targeting.	[159]
PL3	AGRGRLVR	Interacts with the transcytosis receptor NRP-1 to serve as a multifunctional targeting platform for imaging and therapeutic agents in solid tumors.	[160]
SHp	CLEVSRKNG	Exhibits high affinity for glutamate receptors on neurons undergoing oxidative stress–induced apoptosis.	[161]
PVGLIG	PVGLIG	Cleavable by MMP-2 in the tumor microenvironment, enabling potent tumor targeting and prolonged systemic circulation.	[162]
cyclic [RGDKLAK]	c[RGDKLAK]	Improves drug aqueous solubility, enables pH-responsive controlled release.	[164]
Cyclic RGD peptide	RGDfK	Conjugated to DM1 via a disulfide bond for glutathione (GSH)-responsive drug release, with rapid renal clearance and low hepatic accumulation.	[166]

**Table 7:** Motif incorporation for targeting and function.

Category	Peptide Name	Sequence	Targeting Function	Ref.
Targeting ligands				
	RGD	Arg-Gly-Asp	Targeting αvβ3, αvβ5	[192]
	NGR	Asn-Gly-Arg	Targeting aminopeptidase N (CD13)	[193]
	GRP	MTYPRGNHWAVGHLM-NH_2_	Targeting GRPR	[194]
Cell-penetrating				
	TAT48–60	GRKKRRQRRRPPQ	Multiroute cell penetration	[195]
	TAT	YGRKKRRQRRR	Multiroute cell penetration	[198]
	R9	RRRRRRRRR	Direct-route cell penetration	[22]
Nuclear and Mitochondrial targeting				
	Heptadecapeptide	Pro-Lys-Lys-Lys-Arg-Lys-Val	Identify nuclear transporters	[200]
	MTS	MLSLRLLRGLTGSARRLPVPRAKIHSL	Targeting mitochondrial membrane	[201]
Hydrophobic or β-sheet motifs				
	RADA16-I	Ac-(RADA)4-CONH_2_	Self-assembles into β-sheet nanofibers	[119]
	MAX1	VKVKVKVKVDPPTKVKVKVKV-NH_2_	Self-assembled as a hydrogel	[204]
	MAX8	VKVKVKVKVDPPTKVEVKVKV-NH_2_	Self-assembled as a hydrogel	[205]
	Aβsequence	KLVFF	Self-assembles into β-sheet nano-structure	[206]
	Fmoc-FF	Fmoc-Phe-Phe	Self-assembles into nanofibers	[207,208]

## Data Availability

No new data created or analyzed in this study. Data sharing is not applicable to this article.
